# Editorial for Special Issue: Nanoimprint Lithography Technology and Applications

**DOI:** 10.3390/nano11092413

**Published:** 2021-09-16

**Authors:** Michael Muehlberger

**Affiliations:** Profactor GmbH, 4407 Steyr-Gleink, Austria; Michael.muehlberger@profactor.at

Nanoimprint Lithography (NIL) has been an interesting and growing field over the last years since its beginnings in the mid 1990ies. During that time nanoimprinting has undergone significant changes and developments and nowadays is a technology used in R&D labs around the world as well as in industrial production processes. One of the exciting things about nanoimprinting is the big versatility of the process and the broad range of applications it can be used for. This special issue includes 10 articles [1,2,3,4,5,6,7,8,9,10] which represent a small glimpse of the challenges and possibilities of this technology.

Six contributions deal with nanoimprint processes aiming at specific applications [2,4,6,8,9,10], while the other four papers focus on more general aspects of nanoimprint processes [3,5,7] or present novel materials [1,3]. Several different types of nanoimprint processes are used: plate-to-plate [1,2,3,5,7,10], roll-to-plate [4,6], and roll-to-roll [8]. Plate-to-plate NIL here also includes the use of soft and flexible stamps.

Müller et al. [1] report a UV-curable polymer with surface active thiol groups. Such type of materials can open up additional applications and functionalities for nanoimprinting.

In [2] by Haslinger et al. organic thin-film detectors are discussed. A novel device concept is presented making use of a nanoimprinted substrate. The detector is sensitive with respect of the polarization and direction of the incident light.

Aspects of stamp fabrication are addressed in [3] by Marumo et al., including a way of stamp lifetime prediction.

The contribution of Atthi et al. [4] presents the fabrication procedure and properties of superhydrophobic and oleophobic surfaces based on high aspect ratio structures fabricated by roll-to-plate nanoimprinting.

The influence of the initial layer thickness on the nanoimprint process and pattern formation is discussed in detail by Mayer and Scheer in [5] both for thermal NIL as well as for UV-NIL. Guiding charts to choose a convenient initial layer thickness and the theoretical background are presented.

The paper by Prajzler et al. [6] presents the results of roll-to-plate imprinting of optical waveguides for optical interconnect applications and beyond.

The impact of the geometry on the filling properties of the nanofeatures in UV-NIL is discussed in the contribution by Thanner and Eibelhuber [7].

A novel concept of nanoimprint-fabricated micro titer plates for neuronal cells is presented by Lohse et al. in [8]. Roll-to-roll nanoimprinting is used in this paper as a fabrication method.

In [9] Taus et al. describe a fabrication process of master structures for the nanoimprint replication of biomimetic structures, which are inspired by the Morpho butterfly. How these masters are used is described in [10] where it is shown that nanoimprinting is capable of replicating complex undercut nanostructures.

In summary the application fields in this special issue can be identified as plasmonics [2], superhydrophibicity [4], biomimetics [4,9,10], optics/datacom [6], and life sciences [8]. The nanoimprint process related papers discuss filling and wetting aspects during nanoimprinting [3,5,7] as well as materials for stamps [3] and imprinting [1].

I hope that the papers are interesting and entertaining for the readers and that many new ideas will be generated after reading the contributions to this special issue.
